# Molecular surveillance of *pfhrp2* and *pfhrp3* deletions in *Plasmodium falciparum* isolates from Mozambique

**DOI:** 10.1186/s12936-017-2061-z

**Published:** 2017-10-16

**Authors:** Himanshu Gupta, Gloria Matambisso, Beatriz Galatas, Pau Cisteró, Lidia Nhamussua, Wilson Simone, Jane Cunningham, N. Regina Rabinovitch, Pedro Alonso, Franciso Saute, Pedro Aide, Alfredo Mayor

**Affiliations:** 1 ISGlobal, Barcelona Ctr. Int. Health Res. (CRESIB), Hospital Clínic - Universitat de Barcelona, Carrer Rosselló 153 (CEK Building), E-08036 Barcelona, Spain; 2 Centro de Investigação em Saúde de Manhiça (CISM), Maputo, Mozambique; 3 World Health Organization (WHO), Global Malaria Programme, Geneva, Switzerland; 4 Harvard T.H. Chan School of Public Health, Boston, Massachusetts USA; 5 National Institute of Health, Ministry of Health, Maputo, Mozambique

**Keywords:** Malaria, Deletion, RDT, Mozambique, *Pfhrp2*

## Abstract

**Background:**

Malaria programmes use *Plasmodium falciparum* histidine-rich protein-2 (PfHRP2) based rapid diagnostic tests (RDTs) for malaria diagnosis. The deletion of this target antigen could potentially lead to misdiagnosis, delayed treatment and continuation of active transmission.

**Methods:**

*Plasmodium falciparum* isolates (n = 1162) collected in Southern Mozambique were assessed by RDTs, microscopy and/or 18SrRNA qPCR. *pfhrp2* and *pfhrp3* deletions were investigated in isolates from individuals who were negative by RDT but positive by microscopy and/or qPCR (n = 69) using gene-specific PCRs, with *kelch13* PCR as the parasite DNA control.

**Results:**

Lack of *pfhrp2* PCR amplification was observed in one of the 69 isolates subjected to molecular analysis [1.45% (95% CI 0.3–7.8%)].

**Conclusions:**

The low prevalence of *pfhrp2* deletions suggests that RDTs will detect the vast majority of the *P. falciparum* infections. Nevertheless, active surveillance for changing deletion frequencies is required.

## Background

Malarial parasites exhibit striking genetic plasticity that allows their rapid adaption to new drugs [1] and detection methods [2, 3]. This adaptability of the parasite endangers preventive and therapeutic measures against malaria, as the success of control programmes largely relies on early diagnosis and effective treatment. Rapid diagnostic tests (RDTs) are commonly used in malaria case management and elimination programmes, particularly in remote areas where facilities for microscopy are not available [4].

*Plasmodium falciparum* histidine-rich protein-2 (PfHRP2), together with *Plasmodium* lactate dehydrogenase and aldolase, are the key target antigens in commercially available RDTs [5]. Evidence from South America, India and Africa [2, 3] suggest that “deletion” of the target epitope within the parasite PfHRP2 antigen could adversely impact the life of an affected individual as a consequence of delayed or no treatment. Besides *pfhrp2*, *pfhrp3* also affects the performance of RDT, as it has sequence homology with the *pfhrp2* and can be detected by the monoclonal antibodies used against PfHRP2 in RDTs [6].

With increasing false negative RDT reports in African countries, WHO has considered the need of rigorous monitoring of malaria parasites that lack the *pfhrp2* gene [2, 3, 7]. RDTs were introduced in Mozambique in 2007 and national wide use started in 2010 [8]. However, there is no information available about the extent of *pfhrp2* and *pfhrp3* deletions in *P. falciparum* parasites circulating in Mozambique. In this context, this study aimed to assess the presence of *pfhrp2* and *pfhrp3* deletions in *P. falciparum* isolates from Manhiça and Magude districts of Southern Mozambique.

## Methods

### Study site and design

Between 2010 and 2016, a total of 9124 blood samples were collected onto filter papers during cross-sectional studies conducted at the beginning (November) or end (May) of the malaria season in Southern Mozambique (Manhiça and Magude; Table 1). In Mozambique, a peak in transmission is usually seen during the rainy season, from November to April. Transmission intensity in southern Mozambique is generally low, although areas of high transmission may still be observed [9].

**Table 1:** *P. falciparum* isolates collected during cross-sectionals with diagnostic results

Years	Place	Samples collected	*P. falciparum* positive samples	RDT negative, microscopy and qPCR positives samples	RDT negative and only qPCR positives samples
2010	Manhiça	969	105	1	–
2011	Manhiça	842	138	3	–
2012	Manhiça	924	116	3	–
2013	Manhiça	829	166	8	–
2014	Manhiça	908	211	7	–
2015	Manhiça	770	93	9	–
2015	Magude	981	101	7	–
2015	Magude	1322	174	–	125
2016	Magude	1579	58	1	–

Malaria diagnosis was conducted using microscopy, HRP2-based RDT and qPCR; or only RDTs and qPCRs. The inclusion criteria for the deletion analysis were: 1) a negative HRP2-based RDT (SD BIOLINE Malaria Antigen *P. f*—05FK50) but positive by microscopy and qPCR (18S rRNA) or 2) a negative HRP2-based RDT but positive qPCR (18S rRNA) if microscopy was not performed. First, a nested PCR targeting single copy *k13* gene (nPCR_k13_) was performed to verify the presence of parasite DNA in the sample [10]. Second, *pfhrp2* and *pfhrp3* genes were amplified using standard primers as described elsewhere [5, 11]. Finally, *pfhrp2* and *pfhrp3* deletions were concluded if *kelch13* gene PCR was positive, but PCRs for *pfhrp2* and *pfhrp3* failed to amplify the respective gene. The laboratory-adapted culture lines 3D7 as a positive control for both *pfhrp2* and *pfhrp3,* and HB3 and DD2 as negative controls for *pfhrp3* and *pfhrp2,* respectively, were amplified simultaneously. The National Mozambican Ethics Review Committee and the Hospital Clínic of Barcelona Ethics Review Committee approved the collection of samples and molecular analysis. Informed consent and permission (in the case of children under 18 years of age) were also obtained from each participant or a parent/legal guardian during the cross-sectional studies.

### Microscopy

Thin and thick blood smears were air-dried, stained with Giemsa and examined using a light microscope fitted with a 100 × oil immersion lens and a 10 × eyepiece to quantify parasitaemia in the Centro de Investigação em Saúde de Manhiça (CISM) laboratory [9]. Slides were read twice by two different qualified microscopists, and if there was discordance in the results, a third reading was performed by an additional microscopist.

### Rapid diagnostic test

A trained laboratory technician collected approximately 10 μL of blood from an individual by finger-prick to perform an RDT. The PfHRP2-based RDT (SD BIOLINE Malaria Antigen *P. f*—05FK50) was used as per the manufacturer’s instructions.

### DNA extraction and *Plasmodium falciparum* detection by real time PCR (qPCR)

DNA was extracted from a half of the filter paper (Whatman, 903^TM^), containing a 25 μL blood drop by using QIAamp DNA Mini kit (Qiagen). The ABI PRISM 7500 HT Real-Time System (Applied Biosystems) was used to amplify purified parasite DNA templates, using a previously described method [12, 13]. Parasitaemia in the clinical samples was quantified by extrapolation against the standard curve prepared from an in vitro culture of 3D7 strain.

### *kelch13* nested PCR (nPCR_k13_)

Purified DNA templates were amplified using 2720 Thermal Cycler (Applied Biosystems) following a previously described method for the *kelch13* gene [10].

### *pfhrp2* and *pfhrp3* PCRs

Samples with intact parasite DNA confirmed by nPCR_k13_ were used for further amplification of region covering exon 1 and 2, as well as exon 2 of *pfhrp2* and *pfhrp3* genes [5, 11], following previously described methods with minor changes. These changes include the use of 1× HOT FirePol Master Mix, annealing temperatures of 63 **°**C of 1 min for across regions of exon 1 and 2 of *pfhrp2* gene and 60 **°**C of 1 min for exon 2 amplification for both *pfhrp2* and *pfhrp3* genes. PCR products were visualized using 2% agarose (Invitrogen) and a UV trans-illuminator.

## Results

**Fig. 1:**
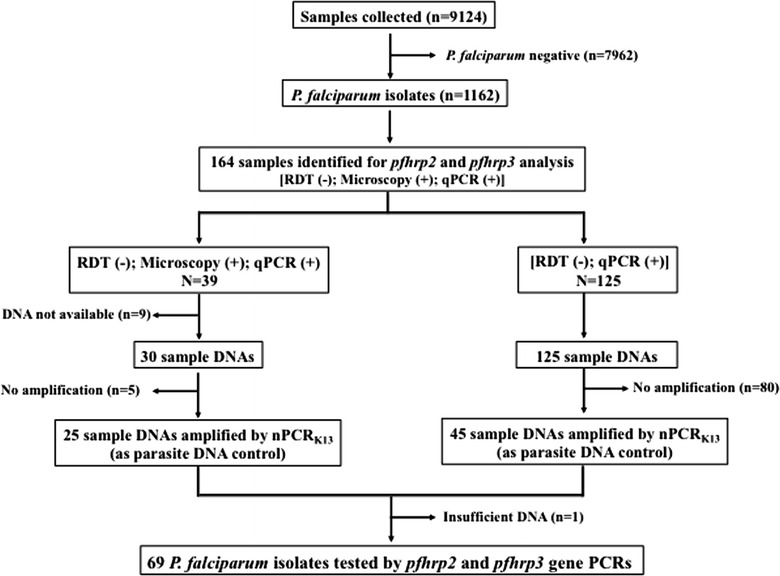
Schematic representation of sample selection for *pfhrp2* and *pfhrp3* deletion analysis

Among the 9124 blood samples collected from participants in cross-sectional studies conducted in Southern Mozambique between 2010 and 2016, 1162 were *P. falciparum* positive by qPCR and/or by microscopy and RDTs. Among these 1162 *P. falciparum* isolates, 164 samples were found eligible for the *pfhrp2* and *pfhrp3* deletion analysis based on a RDT negative, microscopy positive and qPCR positive results (MO+/RDT−/qPCR+; n = 39), or an RDT negative and qPCR positive result (RDT−/qPCR+ ; n = 125). Filter papers and corresponding DNAs were available for 155 (95%) of these 164 *P. falciparum* isolates. Among these, 70 (45%) were positive by nPCR_K13_ (849 bp amplicon size; Fig. 1). Median qPCR parasite densities of the *P. falciparum* isolates that were negative by nPCR targeting *kelch13* gene was 2.17 parasites/µL (interquartile range 1.2–4.4 parasites/µL).

**Fig. 2:**
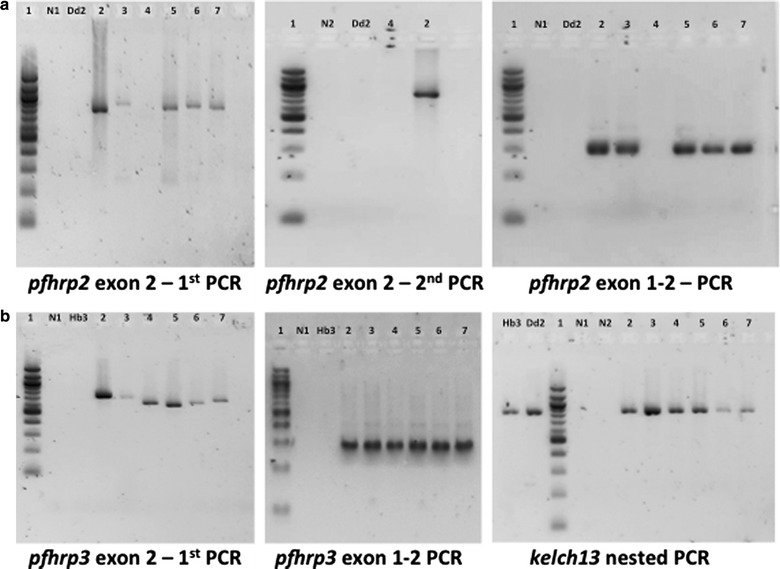
Molecular analysis of *P. falciparum* field isolates along with reference strains 3D7, Dd2 and HB3. **a** Amplification of regions covering exon 1 and 2 (exon 1–2) as well as exon2 of *pfhrp2* in *P. falciparum* field isolates and reference strains. **a** Also show lack of amplification of region exons 1–2 and exon 2 of *pfhrp2* in the field isolate (H1.3), Dd2 strain and negative controls whereas amplification was present in other isolates and 3D7 strain. **b** Amplification of *kelch13* gene, region exon 1–2 and exon 2 of *pfhrp3* in both *P. falciparum* field isolates and reference strains. (Lane 1—100 bp ladder; Lane 2—3D7; Lane 3—H1308; Lane 4—H1.3; Lane 5—H2.4; Lane 6—H2.7; Lane 7—H6.4; N1—negative control first PCR; N2—negative control nested PCR)

69 samples were analysed for *pfhrp2* and *pfhrp3* deletions, as one DNA sample was not enough for the analysis (Fig. 1). Parasite densities in these samples ranged from the 3 to 330,214 parasites/µL by qPCR (Table 2). The laboratory 3D7 strain returned all the expected PCR products of *pfhrp2* (exon 1–2 = 303 bp and exon 2 = 816 bp) and *pfhrp3* (exon 1–2 = 301 bp and exon 2 = 719 bp). As expected, laboratory strains DD2 and HB3 lacked *pfhrp2* and *pfhrp3* amplifications respectively (Fig. 2).

**Table 2:** Parasite densities (parasites/µL of blood), age, sex and year of sample collection of the samples included in the study

Years	Place	Parasitemia by microscopy	Parasitemia by qPCR	Sex	Age (in years)
2010	Manhiça	1670	3858.5	Male	4
2013	Manhiça	56	33.5	Male	12
2013	Manhiça	546	600.3	Male	2
2013	Manhiça	143603	25250	Male	3
2013	Manhiça	203	1500	Male	14
2013	Manhiça	39	13.2	Female	24
2014	Manhiça	232	996.6	Male	4
2014	Manhiça	44594	23469.6	Female	15
2014	Manhiça	140814	84817	Female	3
2014	Manhiça	5721	3827.8	Female	7
2014	Manhiça	52	657.5	Female	3
2015	Manhiça	386	167.58659	Female	14
2015	Manhiça	1657	3407.6235	Female	8
2015	Manhiça	100	98.169426	Female	2
2015	Manhiça	325	156.13457	Male	2
2015	Manhiça	51	32.917961	Male	11
2015	Manhiça	99	108.99004	Male	17
2015	Manhiça	5648	1463.4641	Female	NA
2015	Magude	3610	1610.6819	Female	4
2015	Magude	2950.5	676.75568	Male	9
2015	Magude	303.5	224.56026	Female	50
2015	Magude	928.5	384.57782	Male	2
2015	Magude	2637	95.924171	Female	11
2015	Magude	NA	14.8498	Male	7
2015	Magude	NA	2.44336	Female	10
2015	Magude	NA	27.025	Male	NA
2015	Magude	NA	2.70269	Female	15
2015	Magude	NA	20.8876	Female	47
2015	Magude	NA	330214	Female	3
2015	Magude	NA	99.4996	Female	4
2015	Magude	NA	14.9045	Female	12
2015	Magude	NA	8.46764	Female	7
2015	Magude	NA	9.3634	Male	3
2015	Magude	NA	69.8859	Male	43
2015	Magude	NA	4.21137	Female	17
2015	Magude	NA	4.75359	Female	9
2015	Magude	NA	314.461	Male	4
2015	Magude	NA	104.915	Male	19
2015	Magude	NA	25.1698	Female	11
2015	Magude	NA	27.3208	Male	28
2015	Magude	NA	20.9188	Female	NA
2015	Magude	NA	1184.68	Male	15
2015	Magude	NA	4.68228	Female	9
2015	Magude	NA	612.026	Female	12
2015	Magude	NA	182.307	Female	7
2015	Magude	NA	61.8145	Female	5
2015	Magude	NA	6.33165	Female	2
2015	Magude	NA	50.2857	Female	40
2015	Magude	NA	90.4941	Male	1
2015	Magude	NA	14.7296	Male	3
2015	Magude	NA	539.879	Female	35
2015	Magude	NA	8.85961	Male	12
2015	Magude	NA	231.339	Female	8
2015	Magude	NA	19.9863	Male	15
2015	Magude	NA	55.4191	Female	2
2015	Magude	NA	73.8065	Male	12
2015	Magude	NA	24.4031	Female	29
2015	Magude	NA	419.439	Female	27
2015	Magude	NA	158.95	Male	12
2015	Magude	NA	6.46944	Female	45
2015	Magude	NA	9.88753	Female	40
2015	Magude	NA	2.68125	Male	18
2015	Magude	NA	78.1784	Female	13
2015	Magude	NA	20.7993	Male	15
2015	Magude	NA	576.566	Female	11
2015	Magude	NA	54.7864	Male	9
2015	Magude	NA	19.1635	Female	35
2015	Magude	NA	491.664	Male	45
2016	Magude	609	645.284	Male	31

*NA* not available

No amplification was noticed in negative controls (with water and human genomic DNA), which confirms the *P. falciparum* specificity of all the primer sets used in this study. Expected PCR products were observed upon the amplification of regions across exon 1 and exon 2, as well as exon 2 of *pfhrp2* and *pfhrp3* genes in all the samples except one sample (H1.3). PCR amplification of region covering *pfhrp2* exon 1 and exon 2, as well as exon 2 was not observed in isolate H1.3 (Fig. 2a), while obtaining a positive amplification product for *kelch13* gene. This lack of amplification was confirmed in a second and independent PCR test. The microscopy and qPCR parasite density of sample H1.3 were 2950.5 and 676.75 parasites/µL, respectively. Upon case investigation, this sample was found to correspond to a 30 months old male child from Magude who reported previous episodes of fever (during last 30 days), lived in a fumigated household and slept under a bed net the night before the sample was collected. Apart from this, varying *pfhrp2* and *pfhrp3* exon 2 PCR products lengths (600–1000 bp) were also observed in the analysed samples.

## Discussion

This study provides the first evidence of *pfhrp2* deletion in *P. falciparum* isolates circulating in Southern Mozambique. The prevalence of 1.45% (95% CI 0.3–7.8%) *pfhrp2* deletion among analyzed samples is low as compared to the prevalence observed in other malaria endemic countries such as India (2.4%), Senegal (2.4%), Mali (5%) and Ghana (30.3%) [14–17]. As per WHO guidelines, 5% prevalence of *pfhrp2* deletion has been considered as a minimum threshold to change the type of RDTs [3]. Therefore, PfHRP2-based RDTs are likely to detect the vast majority of the malaria parasites in southern Mozambique, but careful periodic monitoring for changes in deletion frequencies may be required to identify cases such as the single mutant detected in this study.

Previous reports have shown that *pfhrp3* deletion could be an early warning signal for *pfhrp2* deletion [11]. However, the *pfhrp3* deletion has not been observed in the present study. Since only blood spots on filter paper were available in this study, plasma PfHRP2 protein levels or RNA based assays for the same sample could not be performed. However, a number of independent *pfhrp2* PCR based investigation was done to confirm the lack of *pfhrp2* gene in the *P. falciparum* isolate. Moreover, as significant amount of *P. falciparum* isolates (n = 164) were detected by real time PCR but not by nested PCR, given lower sensitivity of the latter [18], and consequently were not eligible for *hrp2/hrp3* assessment. Finally, the varying length of exon 2 of *pfhrp2* and *pfhrp3* PCR products indicates the presence of different numbers of previously identified amino acid repeats [5].

According to 2016 WHO world malaria day fact sheet, the use of RDT has significantly increased globally from 46 million sold in 2008 to 314 million in 2014. In 2014, 53% of global RDTs (*P. falciparum*-specific tests) were delivered to African countries [3]. The excessive use of PfHRP2 based RDTs might enhance the selection of *P. falciparum* isolates with *pfhrp2* deletion, especially in endemic areas where *pfhrp2* deletion is present. Thus, it is important to monitor the presence of parasites with *pfhrp2* and *pfhrp3* deletions to avoid false negative results by RDT. Limitation of the study is that the sample’s material was not available for amplification of flanking genes of *pfhrp2* and *pfhrp3* genes.

## Conclusions

The low prevalence of *pfhrp2* deletions suggests that RDTs will detect the vast majority of the *P. falciparum* infections in Mozambique. However, active surveillance to detect increases in *pfhrp2* deletion frequencies is required towards the common goal to eliminate malaria.
