# Correction to: Molecular surveillance of *pfhrp2* and *pfhrp3* deletions in *Plasmodium falciparum* isolates from Mozambique

**DOI:** 10.1186/s12936-017-2111-6

**Published:** 2017-11-14

**Authors:** Himanshu Gupta, Gloria Matambisso, Beatriz Galatas, Pau Cisteró, Lidia Nhamussua, Wilson Simone, Jane Cunningham, N. Regina Rabinovich, Pedro Alonso, Francisco Saute, Pedro Aide, Alfredo Mayor

**Affiliations:** 1 ISGlobal, Barcelona Ctr. Int. Health Res. (CRESIB), Hospital Clínic - Universitat de Barcelona, Carrer Rosselló 153 (CEK Building), 08036 Barcelona, Spain; 2 Centro de Investigação em Saúde de Manhiça (CISM), Maputo, Mozambique; 3 World Health Organization (WHO), Global Malaria Programme, Geneva, Switzerland; 4 Harvard T.H. Chan School of Public Health, Boston, MA USA; 5 National Institute of Health, Ministry of Health, Maputo, Mozambique

## Correction to: Malar J (2017) 16:416 10.1186/s12936-017-2061-z

After publication of the article [1], it has been brought to our attention that two of the authors have had their names spelt incorrectly in the original publication. The eighth author should be “N. Regina Rabinovich” but was previously spelt as “N. Regina Rabinovitch”. The tenth author should be “Francisco Saute” but was previously spelt as “Franciso Saute”. The original version of this article has now been revised to include these corrections.
